# Immobilization of Penicillin G Acylase on Vinyl Sulfone-Agarose: An Unexpected Effect of the Ionic Strength on the Performance of the Immobilization Process

**DOI:** 10.3390/molecules27217587

**Published:** 2022-11-05

**Authors:** Thays N. da Rocha, Roberto Morellon-Sterlling, Javier Rocha-Martin, Juan M. Bolivar, Luciana R. B. Gonçalves, Roberto Fernandez-Lafuente

**Affiliations:** 1 Departamento de Biocatálisis, ICP-CSIC, Campus UAM-CSIC, 28049 Madrid, Spain; 2 Chemical Engineering Department, Campus do Pici, Federal University of Ceará, Bloco 709, Fortaleza CEP 60440-900, CE, Brazil; 3 Departamento de Biología Molecular, Campus UAM-CSIC, Universidad Autónoma de Madrid, Darwin 2, Cantoblanco, 28049 Madrid, Spain; 4 Department of Biochemistry and Molecular Biology, Faculty of Biology, Complutense University of Madrid, José Antonio Novais 12, 28040 Madrid, Spain; 5 FQPIMA Group, Chemical and Materials Engineering Department, Faculty of Chemical Sciences, Complutense University of Madrid, Complutense Ave., 28040 Madrid, Spain; 6 Center of Excellence in Bionanoscience Research, Member of the External Scientific Advisory Board, King Abdulaziz University, Jeddah 21589, Saudi Arabia

**Keywords:** enzyme immobilization/stabilization, heterofunctional supports, multipoint covalent attachment, immobilization optimization, multi-step immobilization, vinyl sulfone supports

## Abstract

Penicillin G acylase (PGA) from *Escherichia coli* was immobilized on vinyl sulfone (VS) agarose. The immobilization of the enzyme failed at all pH values using 50 mM of buffer, while the progressive increase of ionic strength permitted its rapid immobilization under all studied pH values. This suggests that the moderate hydrophobicity of VS groups is enough to transform the VS-agarose in a heterofunctional support, that is, a support bearing hydrophobic features (able to adsorb the proteins) and chemical reactivity (able to give covalent bonds). Once PGA was immobilized on this support, the PGA immobilization on VS-agarose was optimized with the purpose of obtaining a stable and active biocatalyst, optimizing the immobilization, incubation and blocking steps characteristics of this immobilization protocol. Optimal conditions were immobilization in 1 M of sodium sulfate at pH 7.0, incubation at pH 10.0 for 3 h in the presence of glycerol and phenyl acetic acid, and final blocking with glycine or ethanolamine. This produced biocatalysts with stabilities similar to that of the glyoxyl-PGA (the most stable biocatalyst of this enzyme described in literature), although presenting just over 55% of the initially offered enzyme activity versus the 80% that is recovered using the glyoxyl-PGA. This heterofuncionality of agarose VS beads opens new possibilities for enzyme immobilization on this support.

## 1. Introduction

Enzymes have great relevance in the development of green and sustainable chemical processes because of their high activity under mild conditions, and their high product selectivity and substrate specificity [1,2,3,4,5]. However, enzymes are biological biocatalysts unsuitable for many industrial requirements. The limited stability of many natural enzymes may be improved by different techniques, such as directed evolution [6,7] or site directed mutagenesis [8,9,10], and these tools can also make up for their low activity with substrates far different from their physiological ones [11,12,13,14,15]. Nowadays, researchers may even produce enzymes with an additional artificial active center (the so-called plurizymes), that are biological or not [16,17,18]. Nevertheless, another enzyme limitation is the aqueous solubility of most enzymes, which makes their recovery and re-use complex. The solution to enzyme recovery and reuse was the initial objective of enzyme immobilization [19,20]. Researchers have shown that immobilization may also solve some other enzyme limitations. Proper enzyme immobilization [21] can improve enzyme stability by different methods (prevention of intermolecular interactions, rigidification of the enzyme structure by multipoint covalent attachment, generation of an adequate nanoenvironment, etc.) [22], and also alter enzyme activity, selectivity or specificity, reduce inhibitions or be coupled to enzyme purification [23,24,25,26,27,28,29,30,31]. Immobilization may be a powerful tool in the design of an industrial biocatalyst when adequately utilized [21].

The enzyme penicillin G acylase (PGA) from *Escherichia coli* is one of the first successes of the use of enzymes as biocatalysis in pharma-industry, and is used to produce 6-amino penicillanic acid by hydrolysis of penicillin G [32,33,34]. Curiously, its natural function is still under debate [35]. The enzyme has the potential for use in many other applications, such as thermodynamically or synthetically controlled synthesis of semi-synthetic beta-lactamic antibiotics, resolution of racemic mixtures, and unblocking steps in different synthetic processes, among others [36,37,38,39,40,41,42,43,44]. The enzyme is produced as a monomer that is self-processed with two different subunits [45,46]. The enzyme undergoes a conformation change induced by the interaction with the acyl donor substrate (phenyl acetic or an analog), that exposes the catalytic group to the medium, otherwise the active center is not in contact with the reaction medium [47]. The enzyme has been immobilized on many different supports, and this has proved to be a critical point to widen its range of applications [48,49].

For example, PGA has been utilized in some pioneering works in enzyme immobilization, such as the development of enzyme crosslinked aggregates by the group of Professor Sheldon [50], the use of glyoxyl-agarose [51,52], epoxide-Eupergit [53] or epoxide-Sepabeads [54] supports to stabilize enzymes via multipoint covalent attachment, and the use of site-directed mutagenesis to improve the enzyme immobilization [55,56,57,58] by the group of Professor Guisan. Multipoint immobilized biocatalysts of this enzyme have been utilized to successfully produce antibiotics in the presence of high organic solvent concentrations (conditions where not so stabilized PGA biocatalysts became quickly inactivated) [59,60,61].

However, to date, PGA has not been immobilized/stabilized on supports activated with vinyl sulfone groups. This immobilization support has been recently reported to be very suitable for production of intense enzyme-support multipoint attachment [22,62]. In fact, it can involve not only primary amino groups of the lateral chain of Lys and the protein terminal amino group(s) in the enzyme immobilization, but also thiols, imidazole or phenol groups of Cys, His or Tyr [62], similar to epoxy groups [58,63] but improving the range of reactivity of glyoxyl groups, which only react with primary amino groups [64]. The length of the spacer arm is larger than that of the glyoxyl groups, and this has a double effect [22]. When the spacer arm is longer, it has more mobility and can reach more groups in the enzyme, and can produce more intense multipoint covalent immobilization [62,65]. However, this also has a negative effect, as each enzyme group attached via this longer spacer arm has more mobility, so that each additional bond has a smaller structure rigidification effect. Stabilization compared to glyoxyl supports depends on the specific enzyme, although the number of enzyme-support bonds should be higher [62,65]. As a reaction end point, a blocking step is required to eliminate the chemical reactivity of the support. This permits a final tuning of enzyme features, as the blocking step may determine the enzyme-support interactions, which conditions the final enzyme structure [62,65,66,67,68,69] and the final properties of the immobilized enzyme. This includes not only enzyme stability, specificity and activity [62,65,66,67,68,69], but also the inactivation pathway that the enzyme follows during inactivation [70]. A layer of vinyl sulfone groups is hydrophobic enough to promote the immobilization of lipases on agarose-vinyl sulfone via interfacial activation [65]. These enzymes have a special affinity by hydrophobic supports due to their natural function [71], and this has been exploited to develop specific lipase immobilization protocols [72,73].

The preparation of the immobilization of an enzyme on vinyl sulfone support has at least three steps [59,62,63,64,65,66,67]. The first is the immobilization conditions (usually the immobilization pH value is studied), that are determined by where the enzyme is fixed to the support. This establishes the orientation of the enzyme on the support, and the area that will be involved in the enzyme-support reactions. The second is the incubation conditions, where the already immobilized enzyme reacts with the support, which determines the intensity of the enzyme-support multipoint attachment. The last step is the blocking step, where the remaining vinyl sulfone groups react with different nucleophiles to prevent undesired enzyme-support covalent reactions during operation. This step determines the final enzyme-support interactions [59,62,63,64,65,66,67].

Our aim was to immobilize PGA from E. coli on agarose-vinyl sulfone (VS) and to study the stabilization effects of this immobilization. Agarose beads were chosen because they represent a hydrophilic and inert porous support, and the only groups able to interact with the enzyme should be those introduced by the researcher, in this case vinyl sulfone [74]. Among the strategies to determine enzyme stability, we studied this measuring activity loses under thermal stress conditions, considering that the enzyme was more stable when these activity losses were smaller than those of the reference [75].

## 2. Results

### 2.1. Immobilization of PGA on VS Agarose Beads

**Figure 1:**
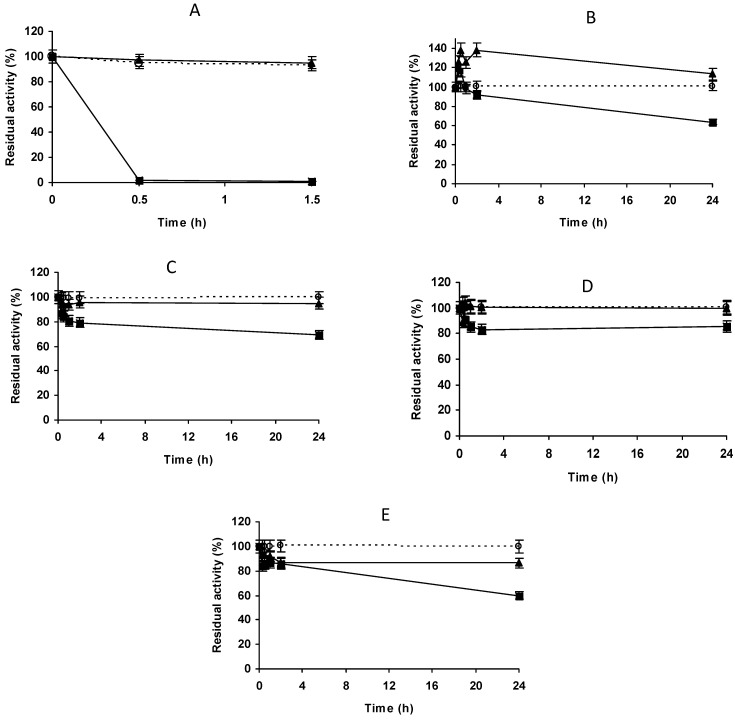
Immobilization courses of PGA (2.5 mg/g) on vinyl sulfone agarose at different pH values. (**A**) Immobilization on glyoxyl agarose support at pH 10; (**B**): immobilization in VS-agarose at pH 7.0; (**C**) immobilization in VS-agarose at pH 8.0; (**D**) immobilization in VS-agarose at pH 9.0; (**E**) immobilization in VS-agarose at pH 10.0. Empty circles with dashed line: reference suspension. Full triangles with solid line: suspension. Full squares with solid line: supernatant. Other specifications are described in Methods.

In an initial trial, we tried to immobilize the enzyme on a VS and glyoxyl support in a ratio of 1/10. This enzyme has been shown to be immobilized very rapidly in glyoxyl agarose [52], even when this support requires the simultaneous establishment of several enzyme-support bonds to fix the enzyme to the support [76]. Figure 1A shows the fast immobilization of PGA on this support (all PGA activity was immobilized in the first activity determination assay over a 30 min period). However, the immobilization on vinyl sulfone-agarose was much slower at all the assayed pH values (including pH 10.0), with immobilization yields under 10%, even after 24 h of immobilization (Figure 1B–E), in contrast to the usual quick immobilization observed with other enzymes [62,65]. This was an unexpected result, as glyoxyl only immobilizes the enzyme via a multi-reaction with several non-protonated primary amino groups [76] and vinyl sulfone can immobilize the enzyme via just one bond, involving many more groups (His, Tyr for example) in the reaction with the support [65]. This agrees with other reports where enzymes that could be immobilized on glyoxyl supports were not immobilized on VS supports [66,67,77], and suggests that immobilization on VS-activated supports may be a more complex process that just the covalent reaction between nucleophiles in the enzyme and the VS groups in the support. In some cases, it was observed that the activity of the suspension slightly increased during the first moments of the immobilization (e.g., at pH 7, Figure 1B), which may be caused by distortions of the enzyme structure with positive effects on the enzyme activity, as observed using this support with other enzymes [65,77].

**Figure 2:**
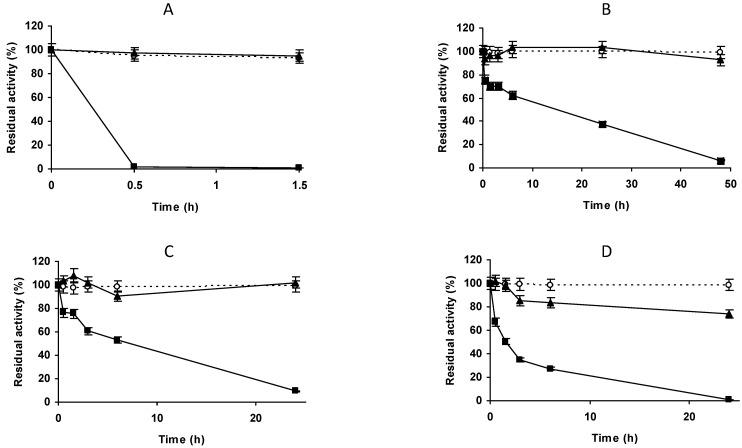
Immobilization courses of PGA (2.5 mg/g) on vinyl sulfone agarose at different pH values in the proportion of 1 g of support for 3 mL of enzyme solution. (**A**) immobilization on glyoxyl agarose support; (**B**) immobilization in VS-agarose at pH 7.0; (**C**) immobilization in VS-agarose at pH 8.0; (**D**) immobilization in VS-agarose at pH 9.0. Empty circles with dashed line: reference suspension. Full triangles with solid line: suspension. Full squares with solid line: supernatant. Other specifications are described in Methods.

**Figure 3:**
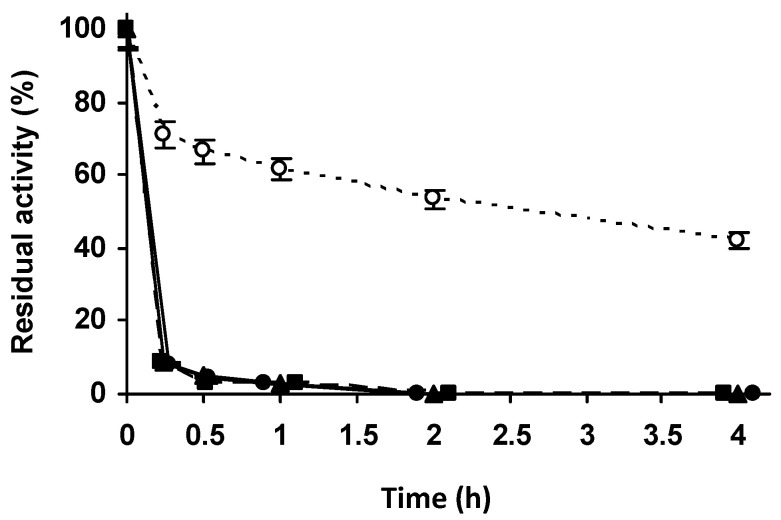
Thermal inactivation courses of PGA immobilized at different pH values. The enzyme was immobilized, in the proportion of 1 g of support to 3 mL of enzyme solution. Incubation was carried out in 50 mM sodium carbonate at pH 9.0 for 3 h and blocked with 2 M glycine at pH 8.0 for 48 h. Empty circles with dashed line: glyoxyl agarose-PGA. Full triangles with solid line: immobilization at pH 7.0. Full squares with solid line: immobilization at pH 8.0. Full circles with solid line: immobilization at pH 9.0. Other specifications are described in the Methods.

To immobilize a higher percentage of enzyme activity and in order to have a high enough activity to check at least the stability of the immobilized enzyme, we concentrated the enzyme and the support using an enzyme solution volume:support mass of 3:1 (Figure 2A–D). Using glyoxyl-agarose, the immobilization remained complete in the first measure (Figure 2A). Under these conditions, the immobilization yield on vinyl sulfone agarose reached a value of 50% after 1.5 h at pH 9, with lower immobilization yields at pH 8 or 7.0 (Figure 2B–D). Immobilization was almost total after 48 h at pH 7.0 or after 24 h at pH 8.0 and 9.0. Although the results were disappointing, the biocatalysts were blocked using Gly and their stabilities were determined. The inactivation courses in Figure 3 show very poor stability of the VS immobilized enzyme when compared to glyoxyl-PGA. All the inactivation courses of vinyl sulfone immobilized PGA are superimposable irrespective of the immobilization condition. This suggests that this strategy of immobilization was not suitable to immobilize or stabilize PGA.

At this point, the hydrophobic character of the VS layer, which created a problem in immobilizing lipases [67], opened up a new opportunity. If this hydrophobicity is high enough to permit the PGA hydrophobic adsorption (at least a partial adsorption) on the support at an ionic strength where the enzyme remains soluble and active, a new opportunity may be opened. The VS-agarose support may behave as a heterofunctional support [66], permitting a first immobilization of the enzyme via hydrophobic interactions, and then permitting the covalent enzyme-support reaction. The use of supports activated with vinyl sulfone and other moieties able to produce the previous adsorption of the enzyme (e.g., octyl-VS, amino VS) have been successful in immobilizing enzymes that do not immobilize on monofunctional VS supports [66,77]. To analyze if the previous adsorption of the enzyme on the support surface could facilitate PGA covalent immobilization on this support, the effect of the ionic strength on the PGA immobilization on VS supports was studied.

### 2.2. Effect of the Ion Strength on PGA Immobilization on Vinyl Sulfone Agarose Beads

**Figure 4:**
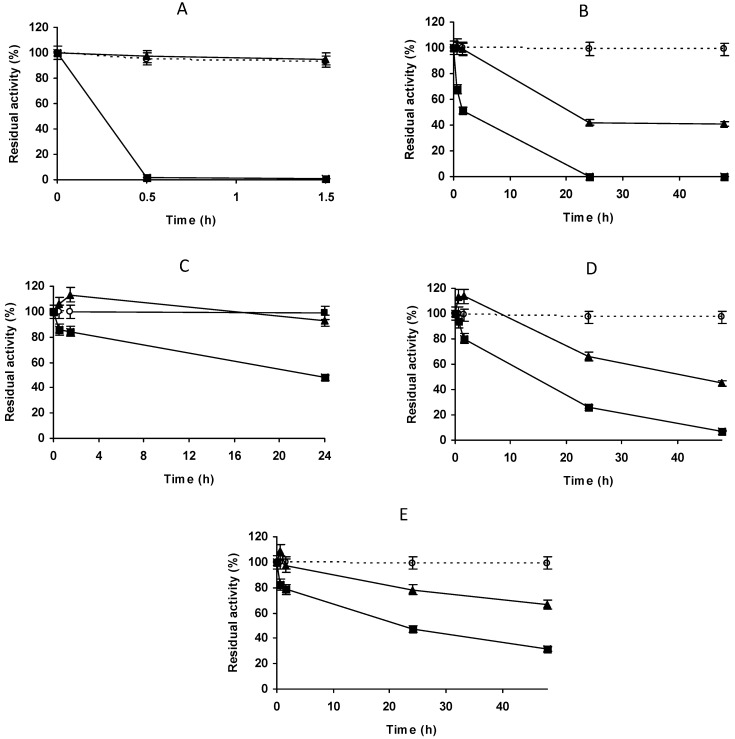
Immobilization courses on glyoxyl agarose (**A**) and on vinyl sulfone agarose at different concentrations of sodium phosphate at pH 8.0, 25 °C. (**B**) 25 mM; (**C**) 250 mM; (**D**) 500 mM; (**E**) 1 M. Empty circles with dashed line: reference suspension. Full triangles with solid line: suspension. Full squares with solid line: supernatant. Other specifications are described in Methods.

**Figure 5:**
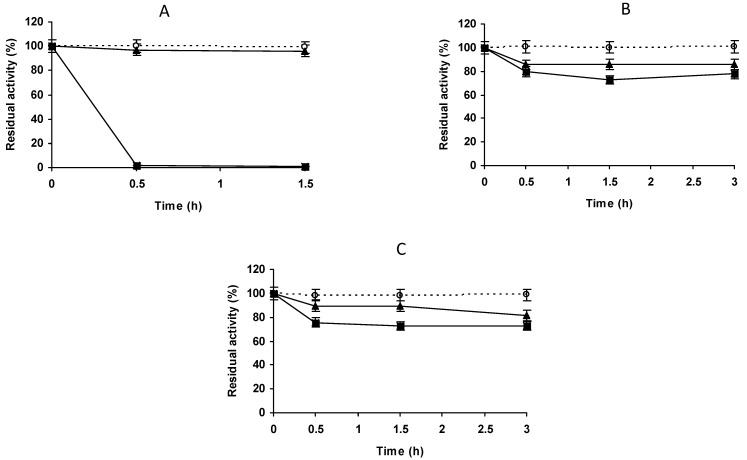
Immobilization courses of PGA (2.5 mg/g) on glyoxyl agarose (**A**) and on vinyl sulfone agarose at different concentrations of sodium chloride in 50 mM sodium phosphate at pH 8.0, 25 °C. (**B**) 500 mM; (**C**) 1 M. Empty circles with dashed line: reference suspension. Full triangles with solid line: suspension. Full squares with solid line: supernatant. Other specifications are described in Methods.

**Figure 6:**
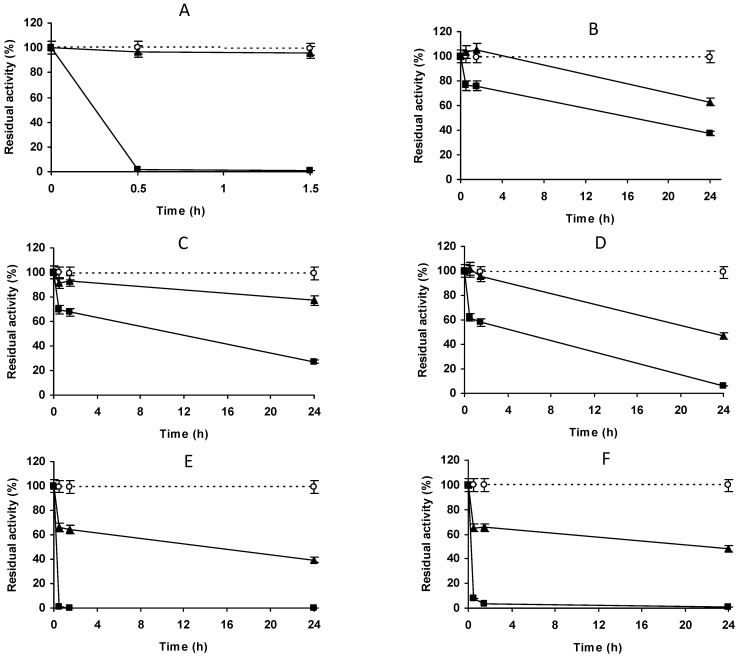
Immobilization courses of PGA (2.5 mg/g) on glyoxyl agarose (**A**) and on vinyl sulfone agarose at different concentrations of sodium sulfate at pH 8.0, 25 °C in 50 mM sodium phosphate at pH 8.0, 25 °C. (**B**) 100 mM; (**C**) 250 mM; (**D**) 500 mM; (**E**) 1 M; (**F**) 2 M. Empty circles with dashed line: reference suspension. Full triangles with solid line: suspension. Full squares with solid line: supernatant. Other specifications are described in Methods.

**Figure 7:**
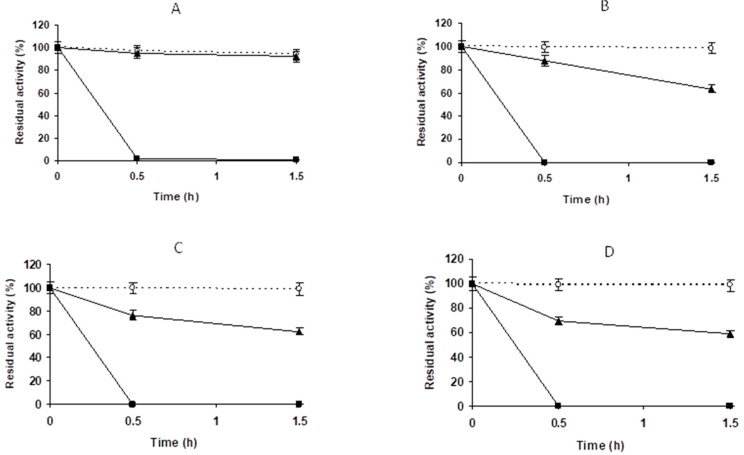
Immobilization courses of PGA (2.5 mg/g) on glyoxyl agarose (**A**) and on vinyl sulfone in 1 M sodium sulfate at 25 °C at different pH values. (**B**) pH at 7.0; (**C**) pH at 8.0; (**D**) pH at 9.0. Empty circles with dashed line: reference suspension. Full triangles with solid line: suspension. Full squares with solid line: supernatant. Other specifications are described in Methods.

Figure 4, Figure 5 and Figure 6 show the immobilization courses of PGA on VS-agarose at different concentrations of sodium sulfate, sodium phosphate and sodium chloride (using a relation of 1/10) at pH 8.0. This pH was chosen because it permits high immobilization rates with other enzymes [62,65,67,68,69,70,77] while it is not deleterious to enzyme stability. The immobilization rate increased with concentration of the salt. One hundred percent immobilization yield was reached after just 30 min employing 1 M sodium sulfate or sodium phosphate, with a less effective immobilization using 1 M NaCl. The figures show how the immobilization rate of the enzyme on glyoxyl-agarose was almost unaffected by the use of 1 M of these salts. Considering the activity of the final biocatalysts and the simplicity of preparing the solutions, further studies were performed using 1 M sodium sulfate. The immobilization of PGA was attempted at pH 7, 8 and 9 on VS-agarose (Figure 7). Under this sodium sulfate concentration, PGA could be fully immobilized on VS-agarose even at pH 7 (Figure 7B). If 0.05% Triton X100 was added, immobilization once again became a very slow process when using VS-agarose (results not shown), confirming that the first hydrophobic adsorption of PGA was required on the support to produce the final covalent immobilization.

The covalent immobilization was so rapid after the PGA adsorption that when we try to release the enzyme from the support, no enzyme could be released from the beginning of the immobilization process (using even detergents).

This result seems to support the hypothesis that the enzyme was first immobilized via hydrophobic interactions at high ionic strength, and then, a covalent reaction between the enzyme and the support was produced. However, when we tried to release enzyme from the support by incubating at low ion strength, no enzyme release could be observed. This could be due to a very rapid enzyme-support covalent reaction after the adsorption at pH 8. In any case, we tried to study the effects of the variables in the different steps on the final features of this biocatalyst.

### 2.3. Effect of Different Experimental Conditions of the Different Steps of the PGA-VS-Agarose Biocatalyst Preparation oniIts Final Features

#### 2.3.1. Effect of the Immobilization pH

Considering that the first step of immobilization seems to be hydrophobic enzyme adsorption on the support, it is possible that the effect of the immobilization pH on the final enzyme performance may be lower than in other cases where this alteration of the reactivity of the enzyme groups with the support may alter the final enzyme orientation [67]. For this purpose, the enzyme was immobilized at pH 7.0, 8.0 or 9.0, maintaining the incubation step at pH 9.0 in the presence of 100 mM phenyl acetic acid/30% glycerol (to minimize enzyme inactivation) [59,78,79] for 3 h and blocking the biocatalyst with Gly. The incubation step was identical for all three biocatalyst with the immobilization pH being the only difference, allowing the same enzyme-support reactivity during the incubation (time and pH value were identical) and using the same blocking agent (that also can affect the final enzyme performance) Table 1 shows the results. In the immobilization step, the activity was higher using pH 7.0 and decreased when increasing the pH (by around one third at pH 9.0). The incubation, even in the presence of protector agents, decreased the activity by approximately an additional 10% for all biocatalysts. The blocking of the biocatalysts with Gly increased the activity of the biocatalysts, very likely by inducing some conformational change of the enzyme that was positive for activity with this substrate.

**Figure 8:**
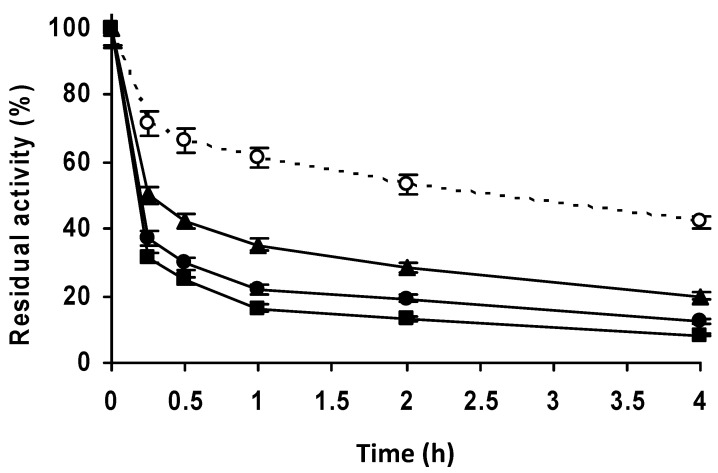
Thermal inactivation courses of PGA immobilized in VS-agarose at different pH values using 1 M sodium sulfate (see legend of Figure 7) compared to the enzyme immobilized in glyoxyl agarose. Empty circles with dashed line: glyoxyl agarose. Full triangles with solid line: immobilization on agarose-VS was performed at pH 7.0. Full squares with solid line: immobilization was performed at pH 8.0. Full circles with solid line: immobilization was performed at pH 9.0. Other specifications are described in the Methods.

Figure 8 shows the inactivation courses of the biocatalysts. They are much more stable than the enzyme immobilized at low ionic strength (Figure 3), but they are still significantly less stable than the PGA-glyoxyl biocatalyst [52,53,54]. Differences among the stabilities of all biocatalysts were not very large, but the enzyme immobilized at pH 7.0 decreased its activity in the slowest fashion, with the fastest inactivation that of the biocatalysts immobilized at pH 9.0. The reactivity of the enzyme with the support during the immobilization step was higher at pH 9.0 and lower at pH 7.0 [62], which could be related to a different orientation of the enzyme on the support and not to a lower number of enzyme-support bonds. Even though the first event of the immobilization seemed to be a hydrophobic adsorption, the immobilization pH played an important role in defining the final enzyme features. Considering activity/stability parameters, immobilization at pH 7.0 was selected as the best condition for this first step.

#### 2.3.2. Effect of Incubation Conditions

It has been shown that high pH value increase the reactivity of enzyme groups with the support, and that a higher intensity of the enzyme supports multipoint attachment [22]. However, enzyme distortions caused by this enzyme-support reaction may result in a milder incubation condition that can yield better activity/stability parameters. PGA was immobilized at pH 7.0, and incubated for 3 h at pH 7.0, 8.0, 9.0 or 10.0 in the presence of phenylacetic acid and glycerol, and finally blocked with Gly. Table 2 shows the activity losses of each step. The incubation at pH 10.0 produced a significant decrease in enzyme activity, down to around 40%, while at pH 9.0 the expressed activity was over 50%, and at pH 7.0 and 8.0, approximately 70%.

**Figure 9:**
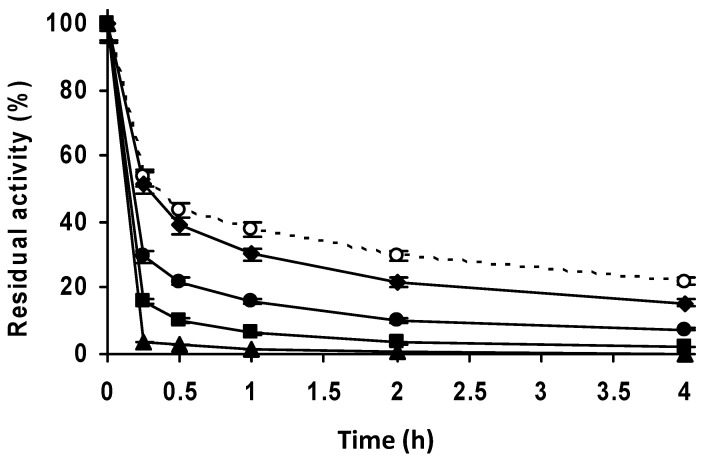
Thermal inactivation courses of PGA immobilized at pH 7.0 in 1 M sodium sulfate and incubated at different pH values (compared to the enzyme immobilized on glyoxyl agarose. The incubation time was 3 h and the biocatalysts were blocked with 2 M glycine. Empty circles with dashed line: glyoxyl agarose-PGA. Full triangles with solid line: sodium phosphate at pH 7.0. Full squares with solid line: sodium phosphate at pH 8.0. Full circles with solid line: sodium carbonate at pH 9.0. Full rhombi with solid line: sodium carbonate at pH 10.0. Other specifications are described in the Methods.

Blocking produced an increase in the enzyme activity that was more significant when the enzyme was incubated at pH 10. Inactivation of the biocatalysts (Figure 9) showed that the most stable biocatalyst was that incubated at pH 10, and was next in stability after the glyoxyl-PGA preparation. The enzymes incubated at the other pH values were less stable when the incubation pH decreased. The activity loss during incubation at pH 10 was partially reversed by the blocking step; therefore, we selected pH 10 as the optimal incubation pH (Table 3). Next, we analyzed the effects of the incubation

**Figure 10:**
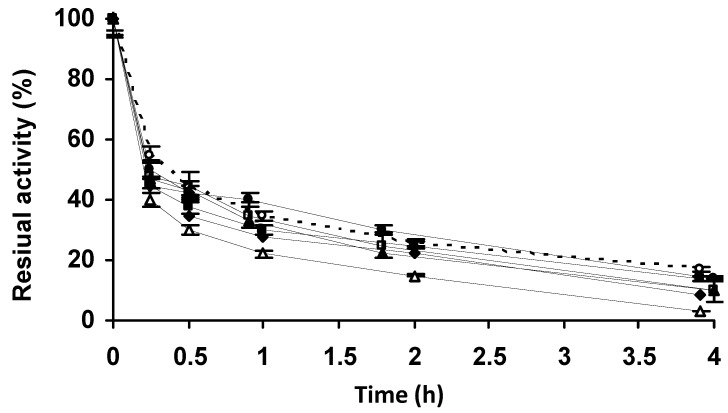
Thermal inactivation courses of PGA immobilized at pH 7 in 1 M sodium sulfate and incubated at pH 10 for different times compared to the enzyme immobilized on glyoxyl agarose The biocatalyst was blocked with 2 M glycine. Empty circles with dashed line: glyoxyl agarose. Empty triangles with solid line: 1 h. Full circles with solid line: 3 h. Full squares with solid line: 5 h. Empty squares with solid line: 24 h. Full rhombus with solid line: 48 h. Full triangles with solid line: 72 h. Other specifications are described in the Methods.

Enzyme-support reaction time is an important parameter in the intensity of multipoint covalent attachment. Immobilization may be just a physical phenomenon (as in this case) or involve just one covalent bond, while to reach the maximum intensity of enzyme-support reaction, it is necessary to leave time lomg enough. Immobilization plays an important role because it involves the reaction between two rigid and not fully complementary structures [21,22,80,81]. Activity decreased from 45% after 1 h to around 25% after 24 h, suggesting a support-enzyme reaction, and remained unchanged later (Table 3). The blocking step partially reversed the negative effects of the incubation time on activity, and increased by almost 1.5 to values ranging 50%. The inactivation rate decerased when going from 1 h to 3 h of incubation (Figure 10), with any later effects being almost negligible. Incubation for 3 h at pH 10 was selected as optimal incubation conditions.

#### 2.3.3. Effect of the Blocking Step

This step alters the enzyme-support physical interactions [70]. Table 4 shows that blocking with Gly and ethanolamine produced the most active preparations (increasing the activity by almost 1.5-fold) and producing a final activity around 55%.

Blocking with β-mercaptoethanol and Asp produced a significant decrease in the activity of the incubated biocatalyst (the final activity was 9% and 3%, respectively), while Cys reduced the activity to 30%. Glucose or ethylenediamine did not affect enzyme activity significantly.

**Figure 11:**
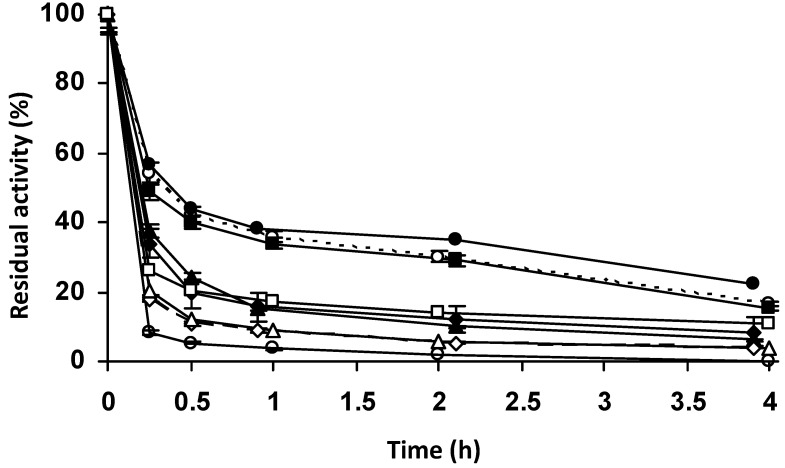
Effect of different blocking agents on the stability of the enzyme penicillin G acylase immobilized at high ionic strength on vinyl sulfone agarose. Immobilization was carried out in 50 mM sodium phosphate pH 7.0 and incubation at pH 10.0. Empty circles with dashed line: glyoxyl agarose as a reference. Full circle with solid line: ethanolamine. Full squares with solid line: glycine. Empty squares with solid line: β-mercaptoethanol. Full rhombi with solid line: cysteine. Full triangle with solid line: control without blocking agent as a reference. Empty triangles with solid line: glucose. Empty rhombi with solid line: ethylenediamine. Empty circles with solid line: aspartic acid. Other specifications are described in the Methods.

Comparison of the stability of different biocatalysts (Figure 11) showed that the unblocked biocatalyst was not the least stable, indicating that an inadequate blocking agent can be negative for enzyme stability. The use of ethanolamine or Gly as blocking agents produced stability similar to that of PGA-glyoxyl (reported to be thousands-fold more stable than the free enzyme) [52,53,54]. Blocking with glucose and ethylenediamine produced biocatalysts slightly less stable than the unblocked biocatalyst, while Cys and mercaptoethanol blocking resulted in stabilities similar to the unblocked biocatalyst. Asp blocking produced a biocatalyst with the lowest stability. Therefore, blocking was found to be decisive for the final stability of the biocatalyst [62,65,67,68,69,70,77], and in this case ethanolamine and Gly produced the best results.

## 3. Materials and Methods

### 3.1. Materials

PGA was purchased from Merck (Madrid, Spain) as an aqueous solution (with a mean value of 86 ± 8 mg of protein per mL and 3.4 U/mg). Phenylacetic acid, 6-nitro-3-(phenyl acetamido) benzoic acid (NIPAB), ethylenediamine (EDA), glycine, ethanolamine, cysteine, glucose, aspartic and β-mercaptoethanol were also acquired from Merck. Sepharose^®^ 4BCL was procured from ABT (Madrid, Spain). Divinyl-sulfone (DVS) was supplied from Thermo Fisher Scientific (Madrid, Spain). The Protein Assay Dye Reagent kit was purchased from Bio-Rad (Alcobendas, Spain). All other reagents were of analytical grade. Glyoxyl-PGA biocatalyst (with an enzyme loading of 2.5 mg/g) was used as reference and prepared as previously described [82]. This biocatalyst is described as among the most stable in the literature [52,53,54]. For this purpose, PGA was diluted in 50 mM sodium carbonate containing 100 mM phenylacetic acid/30% (*v*/*v*) glycerol with the pH adjusted at pH 10.05. Then, glyoxyl-agarose beads were added under gently stirring. After 3 h, solid borohydride to reach a concentration of 1 mg/mL was added, and after 30 min, the biocatalyst was vacuum filtered and washed with 100 mM sodium acetate at pH 5 and with an excess of distilled water using a sintered filter. The immobilization yield was 100% and the expressed activity was 80% [52,53,54].

### 3.2. Methods

#### 3.2.1. Preparation of VS Agarose Beads

Agarose beads were activated by mixing 200 mL of 0.333 M sodium carbonate at pH 11.5 with 7.5 mL of divinyl sulfone under vigorous stirring until a homogeneous solution was obtained [83]. Then, a mass of 10 g of agarose beads was added, and the suspension was subjected to gentle agitation for 2 h. Next, the activated VS support was vacuum filtered with a sintered glass funnel, washed extensively with distilled water and stored at 4–6 °C.

#### 3.2.2. Determination of Protein Concentration

The concentration of a commercial preparation of PGA was determined using the method described by Bradford with some modifications [84]. One milliliter of a 1:5 dilution of Protein Assay Dye Reagent Concentrate was put in a 1 cm width cuvette. Twenty-five microliters of the sample at different dilutions were added and mixed. The solution was incubated for 5 min at room temperature. Then, absorbance was measured at 595 nm. A calibration curve was determined using BSA at different concentrations (0.05, 0.1, 0.2, 0.3, 0.5, 0.7 and 0.9 mg/mL).

#### 3.2.3. Enzyme Activity Assay

A spectrophotometer with magnetic stirring (200 rpm) was used to determine enzymatic activities at a controlled temperature of 40 °C. One unit of enzymatic activity (U) was defined as the amount (µmol) of substrate hydrolysed per minute by the indicated mass of free enzyme or immobilized biocatalyst under the assay conditions. NIPAB was used in the determination of PGA enzymatic activity as described by Kutzbach and Rauenbusch [85]. The substrate was prepared in 50 mM sodium phosphate at pH 7.5 at a concentration of 0.15 mM. Enzyme suspension (100–200 μL) or soluble enzyme was used in the reaction medium to initialize the reaction. Activity was measured following the increase in absorbance at 405 nm caused by the hydrolysis of NIPAB (ε under these conditions is 8730 M^−1^ × cm^−1^).

#### 3.2.4. PGA Immobilization on Vinyl Sulfone Agarose Beads

In all the experiments, PGA was immobilized at a loading rate of 2.5 mg/g of support. A reference suspension was prepared using inert agarose beads (the enzyme was not immobilized on this support). All immobilizations courses were followed by measuring the NIPAB activity in the suspension, supernatant and reference suspension. The activity in the supernatant divided by the activity in the reference suspension gave an accurate measure of the immobilization yield, i.e., the percentage of enzyme that has been immobilized. We calculated immobilization yield (percentage of enzyme immobilized on the support) and expressed activity (observed activity divided by the expected one from the immobilization yield) [86]. The immobilization assays were performed at a ratio of 1 g of support per 10 mL of enzyme solution; if this was changed for any reason, it was mentioned in our report. PGA was diluted in 50 mM sodium phosphate solution at pH 7.0 and pH 8.0, or in 50 mM sodium carbonate solution at pH 9.0. In some instances, some salts (sodium chloride, sodium phosphate or sodium sulfate) were added. After enzyme immobilization, the biocatalysts were filtered and washed with the buffer utilized in the incubation step (50 mM sodium phosphate at pH 7 or 8, or 50 mM of sodium carbonate at pH 9 or 10) and the biocatalysts were resuspended under these conditions for different times to permit multipoint covalently attachment, adding 30% glycerol (*v*/*v*) and 100 mM phenyl acetic acid to prevent enzyme inactivation [59,78,79]. After the desired times, and to put an end to the enzyme-support reaction, the biocatalysts were washed with distilled water and resuspended in 2 M solutions of different nucleophiles (EDA, Gly, ethanolamine, Cys, glucose, Asp or β-mercaptoethanol) in 100 mM sodium phosphate at pH 8.0 for 48 h to block the remaining VS groups in the support. Samples were withdrawn to check enzyme activity during the whole process.

#### 3.2.5. Thermal Inactivation of the Different Biocatalysts

The different biocatalysts were inactivated by incubation in 50 mM Tris-Cl at pH 8.0 in a water bath with the temperature set at 65 ºC. Periodically, samples were taken, and their residual activities were determined using the NIPAB assay described above, considering the initial activity of the preparation as 100% and referencing the activity of the other samples to this initial value as a percentage.

## 4. Conclusions

The direct immobilization of PGA on VS-agarose produced very bad results in terms of immobilization yield, expressed activity and enzyme stabilization. However, VS-agarose has a mild hydrophobic character, making this support a de facto heterofunctional one. The use of high ionic strength enabled rapid PGA immobilization on VS-agarose, initialized by the hydrophobic adsorption of the enzyme on the support, shortly followed by covalent bonding that prevented enzyme desorption. This converted VS-agarose to a heterofunctional support, with the advantages and drawbacks that this can have. The individual study of immobilization, incubation and blocking steps produced PGA biocatalysts with stabilities similar to those of the glyoxyl-PGA agarose, considered among the most stable biocatalysts of this enzyme. The moderately hydrophobic character of VS may be an interesting feature in immobilizing enzymes and proteins that immobilize slowly on VS in a direct way, opening new applications for this support in enzyme immobilization. Moreover, the results show that immobilization of enzymes on VS may follow a somewhat more complex protocol that direct reaction between chemical groups on the enzyme and the VS of the support, as the enzyme immobilized readily on glyoxyl supports but not in VS supports.

## Figures and Tables

**Table 1:** Effect of the immobilization pH on the expressed activity of PGA immobilized on VS-agarose. Biocatalysts were immobilized using 1 M sodium sulfate with 50 mM of each buffer; sodium phosphate at pH 7.0, sodium phosphate at pH 8.0 and sodium carbonate at pH 9.0. Incubation was carried out in 50 mM sodium carbonate at pH 9.0 (100 mM phenylacetic acid and 30% glycerol) for 3 h and blocking with 2 M glycine at pH 8.0. The experiments were conducted as described in the Methods.

Relative Activity (%)			
Biocatalyst (Immobilization pH Value)	Immobilization	Incubation	Blocking
**pH 7**	88.60 ± 4.41	70.50 ± 3.87	75.25 ± 3.26
**pH 8**	77.53 ± 3.87	63.13 ± 3.16	70.32 ± 3.51
**pH 9**	67.56 ± 3.37	57.02 ± 2.85	66.41 ± 2.82

**Table 2:** Effect of the incubation pH on the expressed activity of PGA immobilized on VS-agarose. The enzyme was immobilized at pH 7.0 (see legend of Table 1). Incubation was performed in 50 mM of each buffer (containing 100 mM phenyl acetic acid and 30% glycerol); sodium phosphate at pH 7.0, sodium phosphate at pH 8.0, sodium carbonate at pH 9.0 and sodium carbonate at pH 10.0 for 3 h. Blocking was performed by incubation in 2 M glycine at pH 8.0. The experiments were conducted as described in the Methods.

Relative Activity (%)			
Biocatalyst	Immobilization	Incubation	Blocking
**pH 7**	92.46 ± 4.62	69.37 ± 3.46	75.54 ± 3.13
**pH 8**	85.86 ± 4.29	69.49 ± 3.47	72.66 ± 3.10
**pH 9**	87.51 ± 4.37	55.36 ± 2.96	61.82 ± 2.59
**pH 10**	88.57 ± 4.42	35.33 ± 1.76	52.69 ± 1.88

**Table 3:** Effect of the incubation time on the expressed activity of PGA immobilized on VS-agarose The immobilization was at pH 7 and the incubation was performed in sodium carbonate 50 mM (containing 100 mM phenyl acetic acid and 30% glycerol) at pH 10.0. Blocking was performed by incubation in 2 M glycine at pH 8.0. The experiments were conducted as described in the Methods.

Relative Activity (%)			
Biocatalyst	Immobilization	Incubation	Blocking
**1 h**	71.96 ± 3.59	40.38 ± 1.52	69.14 ± 3.46
**3 h**	90.22 ± 4.51	38.85 ± 1.44	55.08 ± 3.40
**5 h**	80.21 ± 4.01	29.55 ± 1.18	50.35 ± 2.76
**24 h**	70.67 ± 3.53	26.03 ± 1.30	46.42 ± 2.82
**48 h**	83.03 ± 4.15	25.44 ± 1.27	45.95 ± 3.09
**72 h**	79.15 ± 3.95	26.62 ± 1.33	45.90 ± 3.35

**Table 4:** Effect of the blocking agent on the expressed activity of PGA immobilized on VS-agarose. The enzyme was immobilized in 1 M sodium sulfate/50 mM sodium phosphate pH 7.0, incubated in sodium carbonate 50 mM (100 mM phenylacetic acid and 30% glycerol) at pH 10.0 for 3 h. The concentration of the blocking agents was 2 M at pH 8.0 for 48 h. The experiments were conducted as described in the Methods.

Relative Activity (%)			
Biocatalyst	Immobilization	Incubation	Block
**Glycine**	74.79 ± 3.74	40.37 ± 0.72	55.75 ± 1.93
**Ethanolamine**	75.62 ± 3.78	39.67 ± 0.78	57.24 ± 2.86
**Cysteíne**	82.44 ± 4.12	38.95 ± 0.74	30.03 ± 1.50
**Aspartic**	74.32 ± 3.72	40.31 ± 0.76	3.40 ± 0.17
**Ethylenediamine**	76.67 ± 3.83	40.25 ± 0.71	40.05 ± 2.01
**Glucose**	78.91 ± 3.95	41.66 ± 0.78	44.87 ± 2.24
**β-mercaptoethanol**	73.49 ± 3.67	38.02 ± 0.70	9.42 ± 0.47

## Data Availability

Data are available upon requirement from the authors.

## Sample Availability

Samples of the compounds are available from the authors.
